# Performance of the point-of-care circulating cathodic antigen test in the diagnosis of schistosomiasis japonica in a human cohort from Northern Samar, the Philippines

**DOI:** 10.1186/s40249-021-00905-5

**Published:** 2021-09-23

**Authors:** Pengfei Cai, Yi Mu, Kosala G. Weerakoon, Remigio M. Olveda, Allen G. Ross, Donald P. McManus

**Affiliations:** 1 Molecular Parasitology Laboratory, Infectious Diseases Program, QIMR Berghofer Medical Research Institute, Brisbane, Australia; 2 Department of Health, Research Institute for Tropical Medicine, Manila, Philippines; 3 icddr, b, Dhaka, Bangladesh; 4 Present Address: Department of Parasitology, Faculty of Medicine and Allied Sciences, Rajarata University of Sri Lanka, Saliyapura, Sri Lanka

**Keywords:** Schistosomiasis, *Schistosoma japonicum*, Kato-Katz, POC-CCA, ELISA, Droplet digital PCR

## Abstract

**Background:**

Zoonotic schistosomiasis, caused by *Schistosoma japonicum*, remains a major public health problem in the Philippines. This study aimed to evaluate the commercially available rapid diagnostic point-of-care circulating cathodic antigen (POC-CCA) test in detecting individuals infected with *S. japonicum* in a human cohort from an endemic area for schistosomiasis japonica in the Philippines.

**Methods:**

Clinical samples were collectedin 18 barangays endemic for *S. japonicum* infection in Laoang and Palapag municipalities, Northern Samar, the Philippines, in 2015. The presence of CCA in filter-concentrated urine samples (*n* = 412) was evaluated using the commercial kits and the results were converted to images, which were further analyzed by ImageJ software to calculate R values. The diagnostic performance of the immunochromatographic POC-CCA test was compared using the Kato-Katz (KK) procedure, in-house enzyme-linked immunosorbent assays (ELISAs) and droplet digital (dd) PCR assays as reference.

**Results:**

The POC-CCA test was able to detect *S. japonicum-*infected individuals in the cohort with an eggs per gram of faeces (EPG) more than or equal to 10 with sensitivity/specificity values of 63.3%/93.3%. However, the assay showed an inability to diagnose schistosomiasis japonica infections in all cohort KK-positive individuals, of which the majority had an extremely low egg burden (EPG: 1–9). The prevalence of *S. japonicum* infection in the total cohort determined by the POC-CCA test was 12.4%, only half of that determined by the KK method (26.2%). When compared with the ELISAs and ddPCR assays as a reference, the POC-CCA assay was further shown to be a test with low sensitivity. Nevertheless, the assay exhibited significant positive correlations with egg burden determined by the KK technique and the target gene copy number index values determined by the ddPCR assays within the entire cohort.

**Conclusions:**

By using in silico image analysis, the POC-CCA cassette test could be converted to a quantitative assay to avoid reader-variability. Because of its low sensitivity, the commercially available POC-CCA assay had limited potential for determining the status of a *S. japonicum* infection in the target cohort. The assay should be applied with caution in populations where schistosome parasites (especially *S. japonicum*) are present at low infection intensity.

**Graphic abstract:**

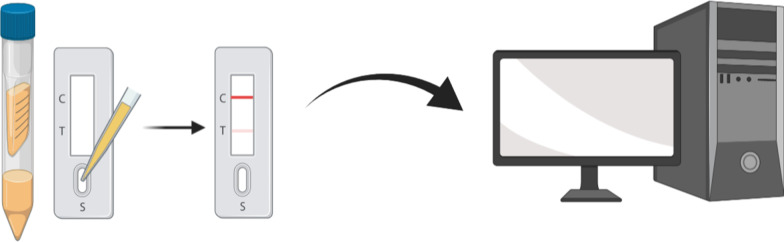

## Background

Schistosomiasis is a debilitating disease caused by agents of the genus *Schistosoma*, which severely affects the health and socio-economic well-being of more than 230 million people in 78 of the world’s poorest countries [1]. In Asia, hepatosplenic schistosomiasis due to *S. japonicum* continues to be of public health concern in the Philippines. Globally, schistosomiasis control focuses on mass drug administration (MDA), in which the effective oral drug praziquantel has served as the cornerstone [2]. In 2013, it was reported that about 860 000 individuals were treated in the Philippines [3]. But the reality is that only a third of those treated actually take the tablets and the estimated cure rate is 52% based on the current treatment regimen, i.e. a single oral dose of 40 mg/kg [4]. As a result, long-term intervention MDA on its own has proven insufficient to provide sustainable control of the disease if no additional integrated interventions are implemented [5]. Affordable and accurate diagnostic tools for rapid mapping and monitoring of schistosomiasis in the context of an integrated control program, including increasing MDA coverage to 85%, and replacing carabaos with mechanized tractors for the tilling in the endemic areas in the Philippines, are urgently needed [6].

There are a variety of methods available for schistosomiasis diagnosis [7, 8]. Parasitological detection procedures [e.g. the microscopy-based Kato-Katz (KK) thick smear and urine filtration methods] exhibit a high level of specificity and these have been recommended as gold standard tests by the World Health Organization (WHO). However, extensive research has now shown that their diagnostic sensitivity is compromised due to the reduced intensity of schistosome infections in this post-MDA era [7]. Improvements to these parasitological-based tests, such as the use of a saline gradient to differentially sediment eggs [9], formalin-ethyl acetate sedimentation-digestion (FEA-SD) for increased visualization of schistosome eggs [10] and Helmintex, which isolates eggs from faecal samples using paramagnetic particles in a magnetic field [11] can increase sensitivity when applied for the detection of intestinal schistosomiasis in endemic areas where there is a low prevalence and egg burdens are reduced; however, these assays are usually time-consuming and labour-intensive. Molecular methods based on polymerase chain reaction (PCR) technology, including real time quantitative (q)PCR- [12–14] and droplet digital (dd) PCR-based tests [15–17], and derivative loop-mediated isothermal amplification (LAMP) [18, 19] are emerging as attractive alternatives for their superior accuracy in the diagnosis of schistosomiasis [20]; however, these procedures need costly equipment and reagents, and skilled human resources, limiting their application in poor rural and marginalised endemic areas.

Antibody detection by ELISA is an alternative approach for schistosomiasis diagnosis and as a powerful screening tool, but it has limited application as a point-of-care (POC) test owing to its limited ability to discriminate past from active infections [21]. Current antigen detection focuses on the probing of proteoglycan components in the gut vomits of juvenile and adult worms known as circulating anodic antigens (CAAs) or circulating cathodic antigens (CCAs) in the format of lateral flow assays or ELISA [1]. The up-converting phosphor-lateral flow CAA (UCP-LF CAA) assay can detect all *Schistosoma* species quantitatively, exhibiting more accurate diagnostic performance than the urine-based point-of-care cathodic circulating antigen (POC-CCA) assay. This assay thus represents a promising tool for the diagnosis of low-intensity schistosome infections [22]. However, UCP-LF CAA is not available as a commercial test as it requires an additional concentration step and a special UCP-LF strip reader [23]. The POC-CCA is a commercially available cassette assay that has been widely validated in the detection of active schistosome infections but the results are determined by visual reading, and the interpretation of a ‘Trace’ reading as ‘positive’ or ‘negative’ is problematical [24].

Evaluation of the diagnostic performance of the POC-CCA test in detecting human infections with the Asian *Schistosoma* species (*S. mekongi* and *S. japonicum*) has not been fully determined. In the present study, we applied a new version of the POC-CCA cassette test for the detection of schistosome antigens in urine samples collected from a human cohort from a moderately endemic area for schistosomiasis japonica in the Philippines two years post-treatment with praziquantel. The results obtained were compared with the KK test and our previously developed in-house ELISA and ddPCR assays that have been tested for the diagnosis of *S. japonicum* infection within the same cohort [17, 25].

## Methods

### Sample collection, processing, and storage

Clinical samples (faeces, blood, urine and saliva) were collected from 412 subjects from 18 barangays in Palapag and Laoang, Northern Samar, the Philippines, in 2015 [17, 25, 26]. Individual stool samples (10–15 g) were collected from each participant with ID-labelled faecal cups. Two faecal samples were sought from each individual on different days within a week for the KK test (3 slides per stool). The remainder of the first faecal sample (~ 10 g) was stored at 4 °C, after fixing in 80% ethanol, and used for DNA extraction. Blood samples (10 ml) were collected from each individual with ID-labeled serum separation tubes (10 ml silica vacutainers). The blood samples were allowed to clot at ambient temperature for 30 min, and serum samples were then collected after centrifugation at 1500 × *g* for 10 min. Saliva (~ 2 ml) was collected into a 5 ml ID-labelled centrifuge tube using the passive drool method under the supervision of a trained medical technologist. All processed samples were stored at 4 °C and transported on wet ice to the Research Institute for Tropical Medicine (RITM) in Manila, where the samples were stored at − 20 °C. All samples were subsequently shipped to QIMR Berghofer Medical Research Institute (QIMRB), Brisbane, Australia on dry ice for analysis. Urine samples from healthy individuals from a non-endemic area in Australia were used as negative controls.

### Parasitological detection (Kato-Katz technique)

KK analysis was performed at RITM. For each stool sample, three KK thick smear slides were made and examined under a light microscope by experienced technicians. Infection intensity was recorded as the number of eggs per gram of faeces (EPG). To ensure adequate quality control of the accuracy of the KK test, 10% of slides were randomly selected for re-examination by an experienced microscopist.

### POC-CCA measurements

**Fig. 1:**
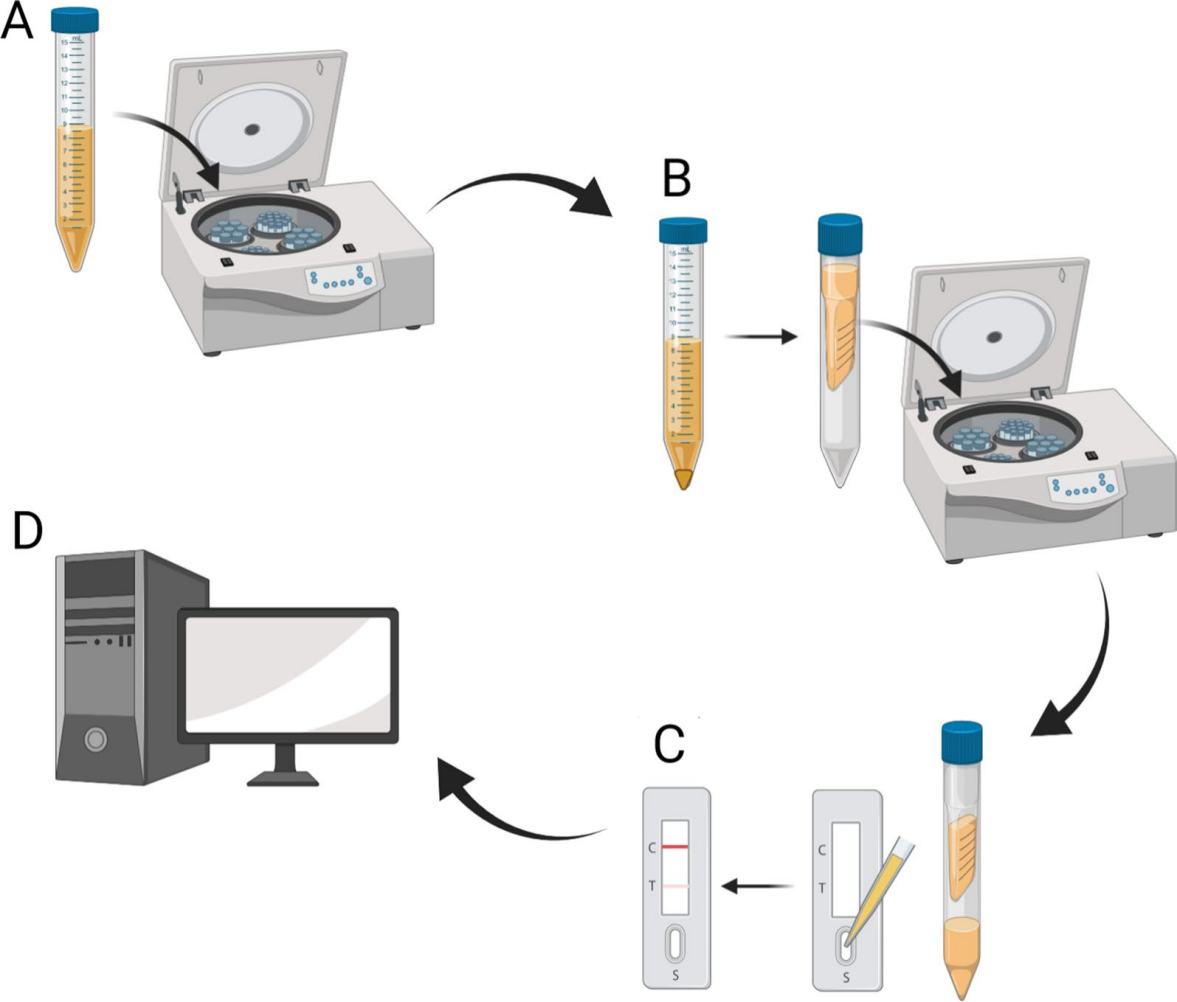
Flow diagram showing the workflow for the detection of *Schistosoma japonicum* infection with the POC-CCA test using samples of concentrated urine. **A** Urine samples were centrifuged to eliminate urinary sediment; **B** Urine supernatants were then concentrated 20 times; **C** The concentrated urine samples were applied to the POC-CCA cassettes and the results were scanned; **D** Images were further analyzed by ImageJ software to calculate R values. Figure 1 was created with BioRender.com

A workflow chart for the detection of *S. japonicum* infection with the POC-CCA test using concentrated urine samples is shown in Fig. 1. Urine samples were left to thaw at 4 °C overnight, then they were mixed thoroughly and centrifuged at 3700 × *g* for 10 min to get rid of urinary sediment. The supernatant was concentrated 20 times using 4 ml single-use 10 kD concentration units (Amicon Ultra Centrifugal Filters, Merck Millipore, Bayswater, VIC, Australia). The presence of CCA in the concentrated urine samples was tested using a commercially available rapid diagnostic kit (POC-CCA, Rapid Medical Diagnostics, Pretoria, South Africa) according to the manufacturer's instructions. Filter-concentrated urine samples (70 µl) were transferred to the circular well of each cassette-based test strip by pipette and the results were read after 20 min. A test was considered invalid if the control band did not appear or if the test was left to develop for more than 25 min, in which case it was repeated. All test images in TIFF format were uploaded to a computer and imported into the Java-based image processing program, ImageJ 1.53 k, to analyse band intensity. The results were transformed to a quantified value by introducing an R value, which was defined as the intensity of the test band divided by the intensity of the corresponding control band within the same cassette.

### Comparative analysis using KK, ddPCR and ELISA assays as references

Genomic DNA isolation of clinical samples (faeces, serum, urine and saliva) collected from the Palapag and Laoang cohort was carried out using the Maxwell 16 Instrument (Promega, Wisconsin) (for faeces) or a ChemagicTM360 instrument (PerkinElmer Inc., Massachusetts) (for serum, saliva, and urine samples) [17]. The purified genomic DNA samples were then subjected to ddPCR and amplification of an 82-bp fragment of the *nad1* gene of *S. japonicum* [16, 17]. The ddPCRs performed on faeces, serum, urine and saliva were designated as F_ddPCR, SR_ddPCR, U_ddPCR and SL_ddPCR, respectively. The ddPCR results are presented as the target gene copy number index (CNI), as previously defined [17]. In addition, two sensitive serum IgG-ELISAs (Sj23-LHD-ELISA, SjSAP4-ELISA), targeting specific *S. japonicum* antigens have been described [25]. Comparative analysis was undertaken on the results of the POC-CCA and the other diagnostic assays (KK, ddPCR and IgG-ELISAs).

### Statistical analysis

All results were entered and stored in a Microsoft Excel 2016 (Microsoft Corp., Seattle, the United States) database. For analysis of differences in the R values between the control group and groups with variable *S. japonicum* egg burdens, one-way ANOVA followed by Holm-Sidak multiple comparison or the Mann–Whitney *U* test was used. A cut-off R value was set for the POC-CCA assay with the maximization of Youden’s *J*-index (defined as *J* = Max_*c*_ {Se (*c*) + Sp (*c*) − 1}). Receiver operating characteristic (ROC) curve analyses were performed and the levels of area under the curve (AUC) were calculated to assess the potential of the POC-CCA test in the diagnosis of schistosomiasis japonica. Differences between the positive rates across the different groups stratified by egg burdens obtained by the POC-CCA and the other diagnostic approaches (the ddPCR assays and ELISA) were tested using McNemar’s test. Differences between the infection prevalences in the endemic cohort determined by the POC-CCA and the other diagnostic methods were tested using McNemar’s test. Compared to the reference tests (KK, ddPCR and ELISA), sensitivity, specificity, positive predictive value (PPV), negative predictive value (NPV) and accuracy were determined for the POC-CCA assay. Agreement analysis between the POC-CCA and the other assays was calculated using the Kappa statistic. The strength of agreement was measured by the Altman’s benchmark scale according to the κ value, with the scores divided into: < 0.20 poor; 0.21–0.40 fair; 0.41–0.60 moderate; 0.61–0.80 good; 0.81–1.00 very good. Pearson’s correlation coefficient (r) was used for the assessment of the correlation between the R values and infection intensity (egg burden; EPG) or CNI values of the ddPCR assays for the whole cohort or the POC-CCA-positives. Statistical analyses were conducted using GraphPad Prism version 7 software (GraphPad Software, Inc., California, USA).

## Results

### Study population and egg burden determined by the KK method

The study population comprised a human cohort (*n* = 412) from a rural schistosomiasis-endemic area, Northern Samar, the Philippines and control subjects (*n* = 15) from a non-endemic area in Australia (Table 1). A total of 108 individuals (26.2%) from the schistosomiasis-endemic area tested positive for *S. japonicum* eggs using six slides from two stool samples with a mean EPG (95% *CI*) of 17.6 (8.8–26.3). Of these KK-positives, the majority (*n* = 104, 96.1%) had a light infection (EPG < 100), and no individuals had a heavy infection (EPG > 400) according to the categorization by WHO on schistosome infection intensity.

**Table 1:** Demographic characteristics and *Schistosoma japonicum* infection intensity of the study subjects in the KK (+), KK (−) and control groups

Group	*n*	Sex (M/F)	Age (years)	Infection intensity (KK)		
				Light (EPG: 1–99)	Moderate (EPG: 100–399)	Heavy (EPG: > 400)
KK (+)	108	70/38	38.9 ± 16.9	104	4	0
KK (−)	304	148/156	40.7 ± 15.2	0	0	0
Control	15	6/9	38.7 ± 16.5	0	0	0

*EPG* Eggs per gram of faeces, *KK* Kato-Katz

### Transforming the POC-CCA test into a quantitative assay by introducing an R value

**Fig. 2:**
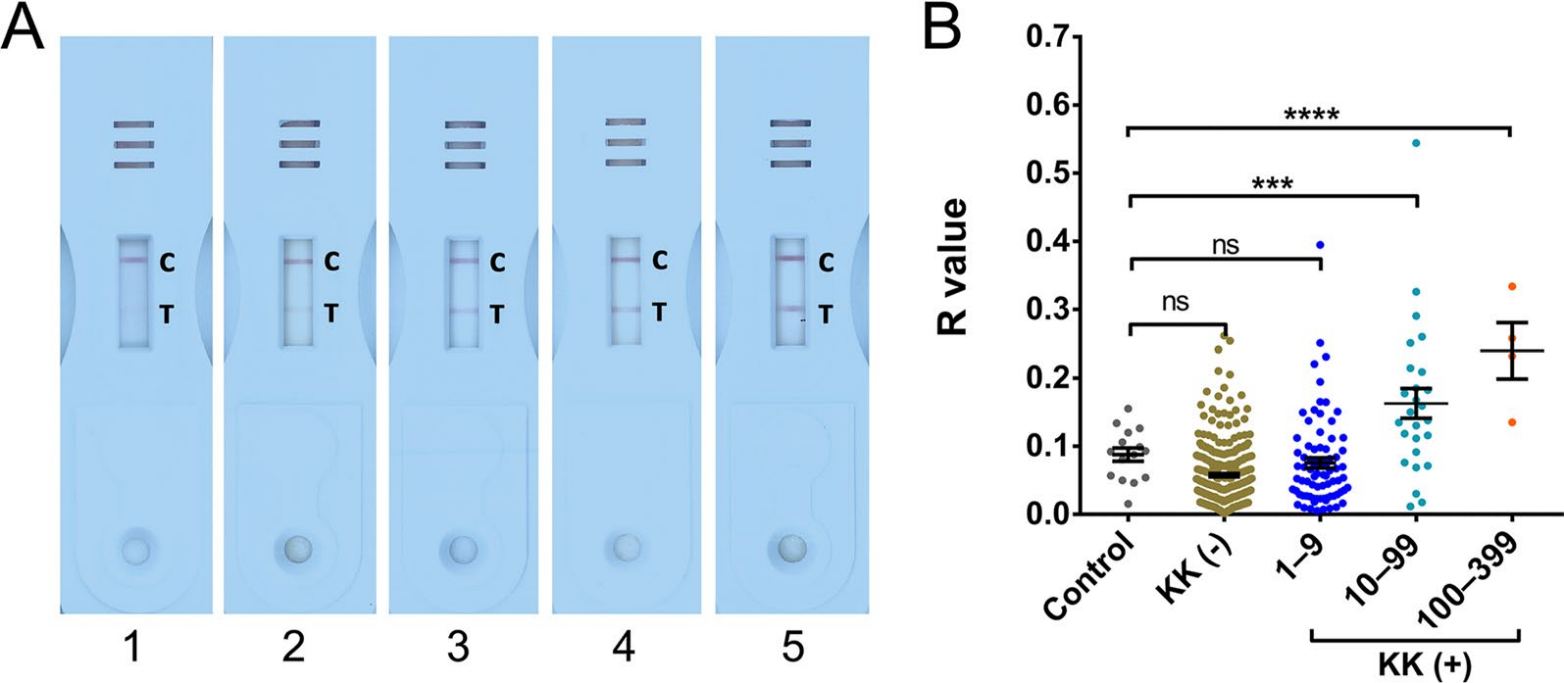
POC-CCA urine cassettes showing different R values and a scatter plot showing the distribution of R values in the different groups stratified by egg burdens and in the control group. **A** POC-CCA urine cassettes showing different levels of the R values. Lanes 1–5, cassettes showing an R value between 0–0.1, 0.1–0.2, 0.2–0.3, 0.3–0.4 and 0.5–0.6, respectively; **B** R values of the POC-CCA tests in controls, KK-negatives and KK-positives with different egg burdens (Control, *n* = 15; KK (−), *n* = 304; EPG 1–9, *n* = 78; EPG 10–99, *n* = 26; EPG 100–399,* n* = 4). *P* values were calculated using One-way ANOVA (ns = no significant difference, *** = *P* < 0.001, **** = *P* < 0.0001). *C* Control line, *T* Test line,  *KK* Kato-Katz,* POC-CAA* Point-of-care circulating cathodic antigen

As indicated in the methods section, for each test cassette, an R value was calculated by dividing the intensity of the test band with that of the corresponding control band. Figure 2A displays POC-CCA urine cassettes showing R values of different ranks. The R values between the control group and the endemic cohort stratified by different egg burdens were further evaluated. The R values of the POC-CCA assays were significantly higher in groups with an EPG of 10–99 (*n* = 26) (*P* < 0.001) and 100–399 (*n* = 4) (*P* < 0.0001) compared with the controls (*n* = 15) (Fig. 2B).

### Diagnostic performance of the POC-CCA urine test using KK (+) subjects as a reference

**Fig. 3:**
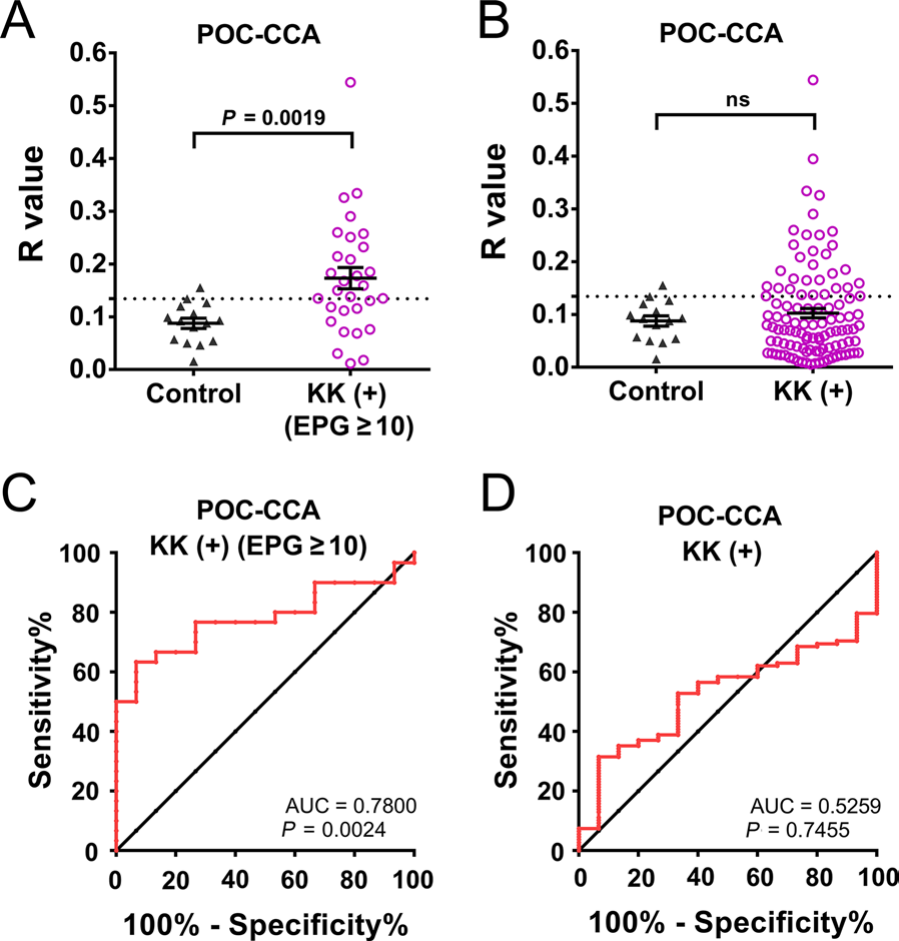
The diagnostic performance of the POC-CCA assay for human schistosomiasis japonica using concentrated urine samples. **A** Healthy control group (*n* = 15) and KK-positives with an EPG ≥ 10 (*n* = 30) were tested by the POC-CCA assay; **B** Healthy controls (*n* = 15) and all KK-positives (*n* = 108) were tested by the POC-CCA assay. *P* values were determined using the Mann–Whitney U test (ns = no significant difference); **C**, **D** ROC curve analysis for the POC-CCA assay was performed to evaluate its capability in discriminating the healthy control group and KK-positives with EPG ≥ 10 (*n* = 30) **(C)** and all KK-positives (*n* = 108) (**D**). *EPG* Egg count per gram, *KK* Kato-Katz, *POC-CAA* Point-of-care circulating cathodic antigen, *AUC* Area under the curve, *ROC* Receiver operating characteristic

The R values of the POC-CCA assay were significantly higher in the KK-positive groups with an EPG ≥ 10 than those of the healthy control group (*P* = 0.0019) (Fig. 3A). No significant difference in the R values was observed between individuals in the control group and the KK-positives with an extremely low egg burden (EPG: 1–9), which comprised the majority (72.2%, 78/108) of subjects (Fig. 3B). By maximizing the Youden’s *J*-index, an R cut-off value was set at 0.1344. The POC-CCA test exhibited 63.3% sensitivity and 93.3% specificity in testing the KK-positives with an EPG ≥ 10. The ROC analysis for discriminating KK-positives with an EPG ≥ 10 from the healthy control group showed that the AUC level for the POC-CCA test was 0.7800 (*P* = 0.0024) (Fig. 3C). The ROC analysis indicated that the POC-CCA test was unable to discriminate all the KK-positives from the healthy control group (*P* = 0.7455) (Fig. 3D).

### Positivity rate and prevalence analysis

All diagnostic tests resulted in a 100% positivity rate in the moderate infection group (EPG: 100–399). In the group with a low egg burden (EPG: 10–99), the positivity rate was significantly higher when determined by the F_ddPCR (100%) (*P* = 0.0026), SR_ddPCR (100%) (*P* = 0.0026), U_ddPCR (88.5%) (*P* = 0.0296) and by the SjSAP4-ELISA (92.3%) (*P* = 0.0159) compared with the POC-CCA test (57.7%). In all subjects with an extremely low egg burden (EPG: 1–9), the positivity rate was significantly higher when determined by the F_ddPCR (97.4%) (*P* < 0.0001), SR_ddPCR (92.3%) (*P* < 0.0001), U_ddPCR (47.4%) (*P* = 0.0004), Sj23-LHD-ELISA (61.5%) (*P* < 0.0001) and SjSAP4-ELISA (91.0%) (*P* < 0.0001) compared with the POC-CCA test (16.7%). The positivity rate with all KK-negatives was significantly lower when determined by the POC-CCA test than with the ddPCR or ELISA assays (*P* < 0.0001, all comparisons) (Table 2). The prevalence of *S. japonicum* infection in the total cohort determined by the POC-CCA test (12.4%) was lower than that obtained with the KK method (26.2%) (McNemar’s test, *P* < 0.0001). The prevalence among the total cohort participants (*n* = 412) was significantly higher when determined by the ddPCR or ELISA assays than by the POC-CCA test (*P* < 0.0001 in all comparisons) (Table 2).

**Table 2:** Positivity rates and prevalences determined by different diagnostic tests across the different groups stratified by parasite burden and in the entire cohort

Diagnostic test	KK (+) Moderate	KK (+) Light^§^				KK (−)		Entire cohort	
	(EPG: 100–399)	(EPG: 10–99)		(EPG: 1–9)		(EPG: 0)		(EPG: 0–399)	
	Positive, % (*n*/*n*)	Positive, % (*n*/*n*)	P*	Positive, % (*n*/*n*)	P*	Positive, % (*n*/*n*)	P*	Prevalence, % (*n*/*n*)	P*
POC-CCA^†^	100 (4/4)	57.7 (15/26)		16.7 (13/78)		6.3 (19/304)		12.4 (51/412)	
F_ddPCR	100 (4/4)	100 (26/26)	0.0026	97.4 (76/78)	< 0.0001	66.1 (201/304)	< 0.0001	74.5 (307/412)	< 0.0001
SR_ddPCR	100 (4/4)	100 (26/26)	0.0026	92.3 (72/78)	< 0.0001	57.6 (175/304)	< 0.0001	67.2 (277/412)	< 0.0001
U_ddPCR	100 (4/4)	88.5 (23/26)	0.0269	47.4 (37/78)	0.0004	43.4 (132/304)	< 0.0001	47.6 (196/412)	< 0.0001
SL_ddPCR	100 (4/4)	76.9 (20/26)	>0.05	23.1 (18/78)	>0.05	20.4 (62/304)	< 0.0001	25.5 (105/412)	< 0.0001
Sj23-LHD-ELISA^#^	100 (4/4)	53.8 (14/26)	>0.05	61.5 (48/78)	< 0.0001	18.1 (55/304)	< 0.0001	30.6 (126/412)	< 0.0001
SjSAP4-ELISA^#^	100 (4/4)	92.3 (24/26)	0.0159	91.0 (71/78)	< 0.0001	56.3 (171/304)	< 0.0001	65.5 (270/412)	< 0.0001

^*ddPCR* Droplet digital PCR; The ddPCRs performed on faeces, serum, urine and saliva were designated as F_ddPCR, SR_ddPCR, U_ddPCR and SL_ddPCR, respectively. *ELISA* Enzyme-linked immunosorbent assay, *EPG* Eggs per gram of faeces, *KK* Kato-Katz; *POC-CCA* Point-of-care circulating cathodic antigen^

^§^Individuals with a light infection were categorised into two subgroups with EPG of 10–99 and 1–9

*P values were determined by McNemar's test

^†^R cut-off value: 0.1344

^#^Cut-off values for ELISA assays: Sj23-LHD-ELISA, 0.2185; SjSAP4-ELISA, 0.1832 [25]

### A comparison of the performance of the POC-CCA test with the other diagnostic methods in detecting of *S. japonicum* infection

Sensitivity, specificity, PPV, NPV, and accuracy were calculated for the POC-CCA assay using the KK, F_ddPCR, SR_ddPCR, U_ddPCR, SL_ddPCR, Sj23-LHD-ELISA and SjSAP4-ELISA separately as the reference test. The POC-CCA assay had the highest sensitivity (29.6%), and accuracy (77.0%) using the KK method as the reference, followed by using the SL_ddPCR as the reference, showing a sensitivity of 27.6% and an accuracy of 76.2%. A similar but low sensitivity (14.3–15.6%) was observed by comparing the POC-CCA test with the F_ddPCR, SR_ddPCR, U_ddPCR and SjSAP4-ELISA assays. The immunochromatographic POC-CCA assay showed a fair agreement (κ = 0.282 and 0.246, respectively) with the KK and SL_ddPCR tests, but had a poor agreement (κ < 0.2) with the other assays (Table 3).

**Table 3:** Performance of the POC-CCA assay using different diagnostic tests as reference

POC-CCA^†^	Reference test		Sensitivity, % (95% *CI*)	Specificity, % (95% *CI*)	PPV, % (95% *CI*)	NPV, % (95% *CI*)	Accuracy, % (95% *CI*)	Kappa index (95% *CI*)
	**+ **	−						
	KK							
+	32	19	29.6 (21.2–39.2)	93.8 (90.4–96.2)	62.8 (48.1–75.9)	79.0 (74.4–83.0)	77.0 (72.6–81.0)	0.282 (0.179–0.385)
−	76	285						
	F_ddPCR							
+	44	7	14.3 (10.6–18.8)	93.3 (86.8–97.3)	86.3 (73.7–94.3)	27.2 (22.6–32.1)	34.5 (29.9–39.3)	0.043 (0.007–0.078)
−	263	98						
	SR_ddPCR							
+	41	10	14.8 (10.8–19.5)	92.6 (86.8–96.4)	80.4 (66.9–90.2)	34.6 (29.7–39.8)	40.1 (35.5–45.2)	0.052 (0.008–0.095)
−	236	125						
	U_ddPCR							
+	31	20	15.8 (11.0–21.7)	90.7 (86.1–94.3)	60.8 (46.1–74.2)	54.3 (49.0–59.5)	55.1 (50.2–60.0)	0.068 (0.001–0.134)
−	165	196						
	SL_ddPCR							
+	29	22	27.6 (19.3–37.2)	92.8 (89.4–95.5)	56.9 (42.3–71.0)	79.0 (74.4–83.0)	76.2 (71.8–80.3)	0.246 (0.142–0.350)
−	76	285						
	Sj23-LHD-ELISA^#^							
+	23	28	22.3 (14.7–31.6)	91.0 (89.4–95.5)	45.1 (31.1–59.7)	77.8 (73.2–82.0)	73.8 (69.3–78.0)	0.160 (0.058–0.261)
−	80	281						
	SjSAP4-ELISA^#^							
+	41	10	15.6 (11.4–20.6)	93.3 (88.0–96.7)	80.4 (66.9–90.2)	38.5 (33.5–43.7)	43.7 (38.8–48.6)	0.068 (0.021–0.114)
−	222	139						

*CI* Confidence interval, *ddPCR* Droplet digital PCR, The ddPCRs performed on faeces, serum, urine and saliva were designated as F_ddPCR, SR_ddPCR, U_ddPCR and SL_ddPCR, respectively. *ELISA* Enzyme-linked immunosorbent assay, *KK* Kato-Katz, *POC-CCA* Point-of-care circulating cathodic antigen, *NPV* Negative predictive value,* PPV* Positive predictive value.

^†^R cut-off value: 0.1344

^#^Cut-off values for ELISA assays: Sj23-LHD-ELISA, 0.2185; SjSAP4-ELISA, 0.1832 [25]

### Correlation analysis

**Fig. 4:**
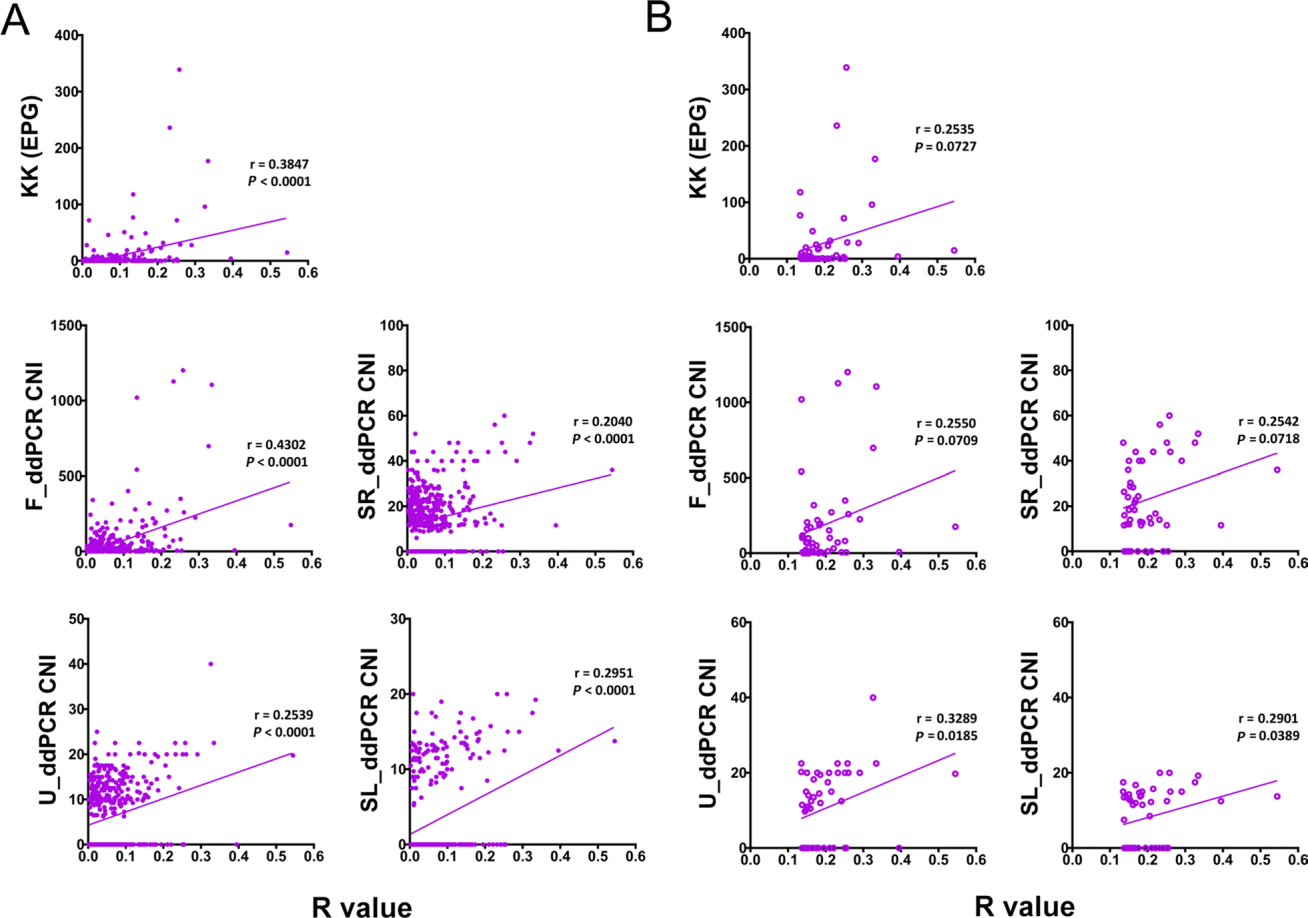
Correlation analysis. Correlations between the R values of the POC-CAA assay and faecal egg burden (EPG) determined by the KK method and CNI values of ddPCR assays in the entire cohort (*n* = 412) **A** and in the POC-CCA-positives (*n* = 51) **B** using Pearson’s correlation coefficient. *CNI* Copy number index, *ddPCR* Droplet digital PCR, *EPG* Egg count per gram, *KK* Kato-Katz, *POC-CCA* Point-of-care circulating cathodic antigen

The associations between the R values of the POC-CCA test and EPG and CNI values were investigated in two groups: the entire study cohort (*n* = 412) and those subjects who were positive by the POC-CCA test (*n* = 51). When analysing the entire cohort, the highest correlation was observed between the POC-CCA and F_ddPCR (r = 0.4302, *P* < 0.0001), followed by the correlation between the POC-CCA assay and KK test (r = 0.3847, *P* < 0.0001) (Fig. 4A). When analysing the POC-CCA positives, significant correlations were only observed between the POC-CCA assay and U_ddPCR (r = 0.3289, *P* = 0.0185) and between the POC-CCA test and SL_ddPCR (r = 0.2901, *P* = 0.0389) (Fig. 4B).

## Discussion

In the current study, we investigated the diagnostic performance of a newly formulated POC-CCA assay by testing urine samples collected from a parasitologically defined Philippines cohort from an area moderately endemic for schistosomiasis japonica. The continuation of high schistosomiasis prevalence in the Philippines is partly due to the lack of affordable, rapid, accurate and field-friendly diagnostic tools necessary for monitoring control efforts. Improved diagnostics, especially the development of inexpensive POC tests are a major focus of the new 2021–2030 roadmap to eliminate neglected tropical diseases recently released by the WHO [27]. Currently, of the diagnostics available, the POC-CCA test best fulfils the ASSURED [A = affordable by the affected individuals; S = sensitive; S = specific; U = user-friendly; R = rapid turn-around time and robust performance (e.g., reagents tolerate tropical climate); E = equipment-free; and D = delivered to those in need] criteria for deployment in the detection of schistosomiasis in resource-limited endemic settings [23]. The POC-CCA test has been widely verified for the rapid diagnosis of schistosomiasis, especially individuals infected with *S*. *mansoni.* The assay has been shown to be satisfactory when applied in schistosomiasis mansoni endemic areas with the KK prevalence below 50%, the prevalence determined by the POC-CCA assay was between 1.5- and sixfold higher than that obtained with the KK method [28].

Accumulating evidence indicates that the reading of the trace results for the POC-CCA test greatly affects the sensitivity of the assay, and is a particular issue in the identification of cases with low parasite burden [29]. When applied in low prevalence areas or endemic settings with low levels of infection intensity, the POC-CCA test can be subjective in balancing diagnostic sensitivity with specificity. If the trace signal results of the POC-CCA test are regarded as positive, this could amount to a considerable level of false positives, whereas sensitivity would be substantially reduced if the trace signal results are considered as negative [30]. For example, the sensitivity of the POC-CCA test dropped from 65.7% to 17.1% in detecting *S. mansoni* infections in a low prevalence area in the Amazon Region of Brazil when the ‘Trace’ results were classified as negative [31]. The reading of the test must be undertaken within 25 min after the sample has been applied to the urine-CCA cassette [32], so as to achieve an accurate diagnostic assessment, any trace result should be verified by a trained investigator. Even so, variability in the reading of a trace result by naked eye can occur due to an individual’s visual acuity and/or the training and experience of the investigator in undertaking the POC-CCA test [24]. Optimization in the interpretation of test results represents the key to accurately reflect the performance of the assay so as to determine the infection status in a cohort where the majority of individuals have a light infection. In the current study, the results of the POC-CCA tests were appropriately scanned and further analyzed in silico to eliminate potential inter-reader variability, a procedure similar to the image analysis undertaken in Tanzania by Casacuberta et al. [33]. Computers and scanners may not be accessible in some endemic settings in the Philippines, therefore impacting the use of the modified POC-CCA method as a field-friendly tool. However, there have been recent significant developments in using smartphone-based point-of-care-testing (POCT) for diagnosis, particularly in the identification of pathogen infections [34–37]. Combining the POCT and POC-CCA procedure can thus provide a novel approach that represents a cost-effective and accurate next-generation diagnostic tool. Furthermore, in the present study, by introducing an R value, we were able to convert the POC-CCA from being semi-quantitative to a fully quantitative method, which can help minimise potential system errors such as varying absorbance levels of urine samples to the test strips among different cassettes.

The issue of diagnostic specificity has been raised as another potential concern with the immunochromatographic assay. A very recently published report showed that the CCA test only had a specificity of 62.1% on testing fresh urine samples collected from individuals residing in a non-endemic area for schistosomiasis mansoni in Brazil [38]. Regarding Asian schistosomiasis, the application of the POC-CCA test in endemic areas for *S. mekongi* in Lao PDR reported that whereas the sensitivity of the POC-CCA assay was only 24.1% yet it was still about two times higher than that of the KK technique (13.6%), when using the overall composite assessment as reference [39]. However, a recent cross-sectional study revealed that other helminths such as *Opisthorchis viverrini* could cause a cross-reaction when the immunochromatographic test was applied in Lao PDR, resulting in false positive diagnosis [40]. Furthermore, this study confirmed previous observations showing the POC-CCA test could result in false positives among subjects with hematuria and urinary tract infections indicating the procedure should be applied with caution as a surveillance tool in areas endemic for *S. mekongi* given the considerable risk of false positive results and the unknown sensitivity of the test [40]. In the endemic area where the current Philippines cohort was recruited, the overall prevalences of human helminthiases including *Ascaris lumbricoides*, *Trichuris trichiura*, *Taenia* spp. and hookworm were at moderate to high levels (48.86, 47.49, 33.79 and 32.88%, respectively), with 70.3% of the rural population in this setting infected with one or more parasitic helminth species [41]. Although the effect of helminth co-infections on the performance of the POC-CCA test is unclear, the studies undertaken in Lao and the Philippines [40, 41] suggest that a relatively high cut-off R value needs to be set to retain a high level of test specificity, which will be an important future consideration as the infection prevalence/intensity is reduced over the course of a schistosomiasis control program [22, 42, 43].

Compared with the Kato-Katz method, lower levels of sensitivity were observed when testing the commercial POC-CCA assay for *S. mekongi* (49% vs 90% of three KK thick smears slides) or *S*. *japonicum* (65% vs 81–91% of a single KK slide) infections in banked urine samples from Cambodia and the Philippines respectively, when the test traces and indecisives were considered as positive [44]. This proof-of-concept study indicated the potential of using urine samples for a CCA-based diagnostic approach for Asian schistosomiasis but that more rational design and optimization would be needed to increase the sensitivity of the assay for detecting *S. mekongi* and *S. japonicum* infections. Previously, it had been shown that the performance of the POC-CCA assay could be improved by using filtration-concentrated urine samples [45]. We adopted a similar step with our aim being to increase the sensitivity of the assay. However, when considering all the KK-positives as a reference, the POC-CCA test performed poorly in detecting *S*. *japonicum*-infections in these subjects (AUC = 0.5259, *P* > 0.05) (Fig. 3D). Furthermore, when using KK-positives with an EPG ≥ 10 as a reference, the assay achieved only a moderate performance in detecting these *S. japonicum-*infected individuals (AUC = 0.7800, *P* = 0.0024) (Fig. 3C). In addition, on comparing the performance of POC-CCA method with other diagnostic methods (Table 3), the *S. japonicum* prevalences estimated by the ddPCR assays were 2–6 times higher than those obtained by the immunochromatographic assay, while the estimated prevalences by the Sj23-LHD-ELISA and SjSAP4-ELISA were 2.0 and 5.3 times higher (Table 2). The infection prevalence determined by the POC-CCA test was lower than that obtained using the KK technique, a method that has been widely shown to be insufficiently sensitive to diagnose schistosomiasis patients with low parasite loads [6, 46]. In addition, a decrease in sensitivity, accuracy and kappa index was observed when using the more sensitive methods as references (Table 3), emphasising the poor sensitivity of the immunochromatographic test in detecting *S. japonicum* infection in the targeted cohort. By analysing the positivity rates of the different diagnostic tests for the detection of *S. japonicum* infection stratified by parasite load, we noted a progressive decrease in the rates determined by the CCA test in the subgroups with EPG 100–399, EPG 10–99, EPG 1–9 to EPG 0, with a considerable drop evident between the subgroups with EPG 10–99 (57.7%) to EPG 1–9 (16.7%) (Table 2). Nevertheless, in terms of detecting an active infection, our study showed that the POC-CCA test positively correlated with faecal egg output for the entire cohort as observed in other studies [44, 47]. In addition, significant positive correlations were found between the POC-CCA and all the ddPCR assays we undertook.

Recently, when used to detect *S*. *mansoni* infection in a low schistosomiasis-endemic area in Northeast Brazil, the prevalence determined by the POC-CCA assay (3.9%) was higher than that obtained by the KK procedure (1.6%) [46]. Here, the prevalence of *S*. *japonicum* infection in the total cohort based on the immunochromatographic test (12.4%) was only about half of that determined by the KK method (26.2%), although concentrated urine samples were used for the test. This can be explained by the following reasons: (1) The adult worm is the main source of the circulating cathodic antigen [48] and based on results with the experimentally infected baboon model, CCA is only detectable in infections of 50 worms or more [32]. However, there is a considerable difference in the egg production by paired adult worms as female worms of *S. japonicum* can produce up to 3000 eggs per day, ten times more than *S*. *mansoni*. Consequently, a significantly smaller number of *S. japonicum* worm pairs would produce a similar faecal egg burden as *S. mansoni* in an infected mammalian host. (2) According to parasite load (determined by the KK technique), the Philippines study cohort was characterized as having moderate *S*. *japonicum* prevalence and low infection intensity. Positivity analysis showed the poorer sensitivity of the POC-CCA test could be justified by the very low egg counts per gram faeces (< 10). (3) The capturing antibody (a monoclonal Ab) spread on the test line of the immunochromatographic cassettes was developed against the CCA of *S*. *mansoni*. The affinity of the antibody to the CCA originating from a species other than *S. mansoni* (in this case, *S. japonicum*) may thus decrease. In this respect, it has been reported that the performance of the POC-CCA assay in the diagnosis of *S. haematobium* and *S. mekongi* is also inferior to the detection of *S. mansoni* infection [29, 44]. (4) A relatively high R cut-off value was set to obtain a higher level of specificity for the CCA assay; this is important for the detection of low intensity infections, particularly in the application of the test for large-scale monitoring.

## Conclusions

The commercial POC-CCA assay was able to identify *S. japonicum*-infected subjects in a Philippines cohort with an EPG greater than or equal to 10 with a sensitivity of 63.3% and a specificity of 93.3%. However, the test was unable to discriminate individuals, with an EPG less than 10, from uninfected controls, showing it exhibited low sensitivity in detecting all KK-positive subjects. A comparison of the diagnostic performance of the POC-CCA assay with other diagnostic methods (ddPCR assays and ELISA tests) targeting the whole cohort further revealed the immunochromatographic test was insufficiently sensitive. The POC-CCA assay in its present form is unsuitable for application as a cost-effective tool for the detection of individuals with low *S. japonicum* infection loads in rural schistosomiasis-endemic areas; this outcome reinforces the necessity of developing a more accurate POCT kit for the control of this disease, especially when elimination is the goal. Nevertheless, improvements to the method described here to increase the accuracy of the POC-CCA assay results could be employed as an optimization step for other antigen-detection based POC tests developed for the detection of schistosome infections and other pathogens such as COVID-19.

## Data Availability

Data is available from the corresponding author upon reasonable request.

## Abbreviations

CAA: Circulating anodic antigen
CCA: Circulating cathodic antigen
*CI*: Confidence interval
CNI: Copy number index
ddPCR: Droplet digital PCR
ELISA: Enzyme-linked immunosorbent assay
EPG: Egg count per gram
KK: Kato-Katz
MDA: Mass drug administration
NPV: Negative predictive value
POC: Point-of-care
PPV: Positive predictive value
UCP-LF: Up-converting phosphor-lateral flow
WHO: World Health Organization
